# A Highlight of the Mechanisms of Immune Checkpoint Blocker Resistance

**DOI:** 10.3389/fcell.2020.580140

**Published:** 2020-12-04

**Authors:** Qian Huang, Yanna Lei, Xiaoying Li, Fukun Guo, Ming Liu

**Affiliations:** ^1^Laboratory of Signal Transduction and Molecular Targeted Therapy, Department of Medical Oncology, West China Hospital, Sichuan University, Chengdu, China; ^2^Division of Experimental Hematology and Cancer Biology, Children’s Hospital Medical Center, Cincinnati, OH, United States

**Keywords:** immunotherapy resistance, PD-1, anti-PD-1, immune microenvironment, ICB

## Abstract

In recent years, as our understanding of tumor immunology is continuously improved, immunotherapy has come to the center stage of cancer therapy and is deemed as the most promising approach for cancer control. Although immunotherapy, particularly immune checkpoint blockade (ICB), has achieved a milestone in several types of tumors, the majority of cancer patients do not benefit from immunotherapy. The dismal outcome of cancer immunotherapy is mainly due to primary or acquired resistance arising from tumor immune evasion. Exploring the mechanisms of tumor immune evasion in the course of immunotherapy may identify biological targets to conquer tumor resistance to immunotherapy. In this review, we highlight tumor cell-intrinsic and -extrinsic factors that may underlie tumor resistance to immune checkpoint blockers. Targeting these factors in combination with immune checkpoint blockers points to the future direction of cancer immunotherapy.

## Introduction

Upon the recognition of tumor antigens presented by MHC-I molecules, T lymphocytes are activated to infiltrate into the tumor microenvironment to inhibit and kill tumor cells. However, tumor cells exploit numerous approaches to evade T cell-mediated killing. One such approach is to upregulate immune checkpoints, a group of molecules that repress the activation and function of immune cells including T cells. Immune checkpoints promote self-tolerance in physiological environment and immune evasion in malignant environment. Immune checkpoint blocking is the most promising research direction in the field of tumor immunotherapy. Anti-programmed cell death protein-1 (Anti-PD-1)/Anti-programmed cell death ligand-1 (Anti-PD-L1) or Anti-cytotoxic T lymphocyte associated protein 4 (Anti-CTLA-4) antibodies are currently attractive anti-cancer immune checkpoints blockers (ICB). Ipilimumab (Anti-CTLA-4), Pembrolizumab, Nivolumab (Anti-PD-1), and Atezolizumab, Durvalumab, Avelumab (Anti-PD-L1) have shown significant efficacy in clinical and clinical trials, and have been approved for treating a number of cancers. However, cancer patients often develop primary or acquired resistance to ICB and immune-related adverse events (IrAE). Therefore, a comprehensive exploration of the causes of immune drug resistance will help identify biological targets to conquer drug resistance of cancers to ICB (Syn et al., 2017; No authors listed, 2019).

**FIGURE 1:**
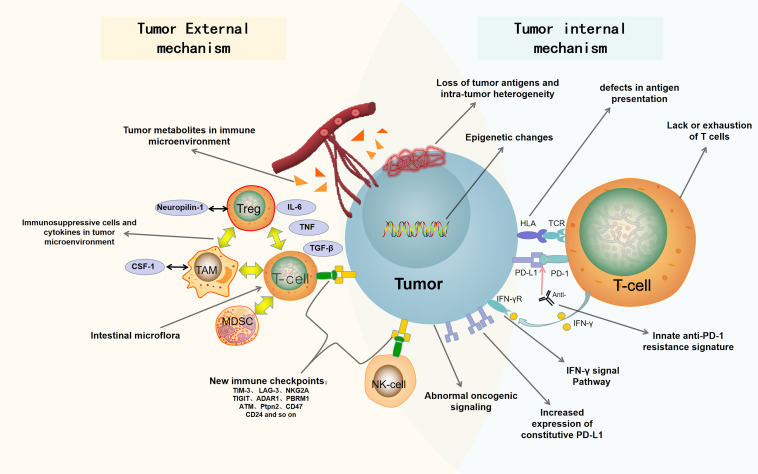
Summary of drug resistance in tumor immunotherapy. The mechanisms of drug resistance and immune escape in tumor immunotherapy include tumor cell-internal and -external factors. The internal factors include loss of tumor antigens and intratumor heterogeneity, defects in antigen presentation, abnormal oncogenic signaling and IFN-γ signal pathway, innate anti-PD-1 resistance signature, increased expression of constitutive PD-L1, epigenetic changes, and lack or exhaustion of T cells. The external factors include immunosuppressive cells and cytokines in tumor microenvironment, tumor metabolites in immune microenvironment, new immune checkpoints, and intestinal microflora. PD-1, programmed death 1; PD-L1, programmed death ligand 1; Anti, Anti-PD1/anti-PDL1; HLA, human leukocyte antigen; TCR, T-cell receptor; IFN-γ, interferon-γ; IFN-γR, Interferon-γ receptor; Treg, regulatory T cell; Neuropilin-1, neurociliary protein 1; TAM, tumor associated macrophage; CSF-1, macrophage colony stimulating factor; MDSC, myelogenous suppressor cell; IL-6, interleukin-6; TGF-β, transforming growth factor-β; TNF, tumor necrosis factor.

In this review, we summarize the recent findings of internal and external causes of tumor resistance to ICB. The internal causes focus on the inherent characteristics of tumor cells, such as tumor antigenicity, tumor escape mutation, interferon signal pathway, epigenetic, carcinogenic signal pathway, and so on; the external causes are mainly emanated from the tumor microenvironment, such as immunosuppressive cells, cytokines, metabolites, new immune checkpoints, intestinal microorganisms, and so on (Pitt et al., 2016; Kalbasi and Ribas, 2020). Figure 1 is a summary of the mechanisms of tumor resistance to immunotherapy. We also discuss the latest research progress in overcoming tumor resistance to ICB, in which combined immunotherapy stands at the center stage.

## Internal Mechanisms of Tumor Resistance to Immunotherapy

### Loss of Tumor Antigens and Intra-tumor Heterogeneity

A neoantigen is an antigen encoded by the mutant gene of tumor cells. It is cross-presented to T cells by dendritic cells (DCs), which can promote T cells to proliferate and become mature activated T cells that specifically recognize the neoantigen. There are more and more evidences that neoantigens are effective targets of immunotherapy, and there is a correlation between tumor mutation burden (TMB) and the efficacy of ICB. For example, based on complete exome sequencing of tumor cells from 42 non-small cell lung cancer (NSCLC) patients who developed acquired drug resistance after single PD-1 or PD-1 combined with CTLA-4 blocking therapy, it was found that the occurrence of acquired drug resistance was related to the loss of neoantigens, not only by eliminating tumor subclones, but also by chromosomal loss of the trunk (Anagnostou et al., 2017). In a responder of NSCLC patients treated with Pembrolizumab, whole exon sequencing showed that neoantigen-specific CD8^+^ T-cell responses were parallel to tumor regression, indicating that Anti-PD-1 therapy enhances tumor neoantigen-specific T cell responses (Rizvi et al., 2015). Mismatch repair defect (MMR-d) tumors are characterized by microsatellite sequence changes, which can accumulate thousands of mutations and result in continuous renewal of neoantigens *in vitro* and *in vivo*. This high mutation burden makes the tumor immunogenic and sensitive to PD-1 inhibitors. After 22 patients with metastatic colorectal cancer and microsatellite instability-high (MSI-H) were treated with PD-1/L1 inhibitors, the objective response rate (ORR) and progression-free survival (PFS) of patients with high TMB were higher than those of patients with low TMB (Germano et al., 2017; Mandal et al., 2019; Schrock et al., 2019). These data support the important role of neoantigens in anti-tumor immune response, so the loss of tumor neoantigens may make tumors less sensitive to ICB. However, it has recently been found that patients with high TMB respond differently to immunotherapy, and many patients with low TMB respond to immunotherapy. An analysis of melanoma patients in TCGA database found that TMB and gene copy number variants, as a single predictor, were not associated with survival. Nonetheless, the survival time of patients with low tumor heterogeneity was significantly better than that of patients with high heterogeneity. Among them, the patients with high heterogeneity and low TMB had the worst survival rate. The researchers sequenced the whole exon of a melanoma cell line irradiated with moderate ultraviolet-B (UVB), and found that TMB and heterogeneity of the irradiated melanoma cells increased. Cancer cells exposed to ultraviolet radiation had little response to PD-1 inhibitors. This study shows that tumor heterogeneity in combination with TMB is a good marker for predicting the efficacy of PD-1 inhibitors in treating melanoma (Wolf et al., 2019).

### Defects in Antigen Presentation

The increase of somatic mutation rate of human leukocyte antigen (HLA) genes is significantly related to the abnormal function of HLA, which is the potential mechanism of immune escape and participates in tumor formation and tumor progression. Effective tumor antigen presentation to CD8^+^ T cells relies on class I HLA (HLA-I). Tumor cells could lose their ability to present neoantigens due to the loss of HLA, which may promote tumor immune escape. The anti-tumor effect of ICB depends on CD8^+^ T cells, which is HLA-I-dependent immune response. In a two-cohort study, more than 1,535 patients with different tumor types were treated with ICB. It was found that patients with all heterozygous HLA loci, no heterozygous loci and high mutation load had higher survival rates. The loss of heterozygote at HLA locus and low tumor mutation load were not conducive to the efficacy of ICB. HLA-B44 is a subtype of HLA, which can cross-present neoantigens presented by other subtypes of HLA and increase the diversity of HLA in disguise. The survival rate of patients with HLA-B44 positive and high mutation level was significantly better than that of HLA-B44 negative patients with low mutation level (Chowell et al., 2018). Loss of heterozygosity in human leukocyte antigen (LOHHLA), a computational tool developed to determine HLA allele-specific copy number from sequencing data, was used to identify HLA LOH in 90 patients with NSCLC. It found that 40% of the patients had loss of heterozygosity in HLA, which impaired their ability to present antigens. As a result, patients with HLA LOH were less likely to benefit from immunotherapy (Gettinger et al., 2017; McGranahan et al., 2017). In addition, analysis of recurrent biopsy samples from four patients with metastatic melanoma treated with Ant-PD-1 revealed a truncated mutation in the gene of antigen presentation protein β2-microglobulin (B2M). This mutation resulted in the loss of MHC I expression on the cell surface (Zaretsky et al., 2016). In summary, the loss of heterozygosity of HLA and the mutation of B2M dampen the processing and presentation of antigens, resulting in the inability of CD8^+^ T cells to recognize tumor antigens and specifically kill tumor cells, which represents an important mechanism of primary or acquired resistance to ICB therapy.

### Abnormal Oncogenic Signaling

Cancer is caused by various genetic changes that cause abnormalities in carcinogenic signaling pathways. More and more evidences show that the activation of carcinogenic pathways in tumor cells can impair the induction or execution of local anti-tumor immune response (Spranger and Gajewski, 2018). In the following paragraphs, we will focus on the effects of crucial oncogenic signal pathways for tumor immune escape, such as Wnt/β-catenin signal pathway, Phosphatase and tensin homolog (PTEN) signal pathway and Mitogen-activated protein kinase (MAPK) signal pathway (Kobayashi et al., 2020).

#### Activation of Wnt/β-Catenin Signal Pathway

The typical Wnt/β-catenin signal pathway is involved in the regulation of gene transcription and participates in organogenesis and tumorigenesis. The activation of Wnt/β-catenin signal can affect immunotherapy, leading to T cell rejection and tumor resistance to anti-PD-L1/anti-CTLA-4 monoclonal antibodies. In the study of metastatic melanoma, it was found that the activation of β-catenin inhibited the production of CCL4, which inhibited the recruitment of BATF3^+^ DCs into the tumor microenvironment. β-catenin activation also inhibited CXCL9/10 production from CD103^+^ DCs, resulting in a suppression of effector T cell infiltration into the tumor microenvironment (Spranger et al., 2015, 2017). In a genetically engineered mouse model of liver cancer, it was indicated that the activation of β-catenin promoted immune escape and resistance to Anti-PD-1 therapy, and weakened the activity of T cells through blockade of recruitment of DCs (Ruiz de Galarreta et al., 2019).

#### Deletion of PTEN Suppressor Gene

In patients with melanoma, PTEN deletion in tumor cells increases the expression of immunosuppressive cytokines and reduces the infiltration of T cells, which likely accounts for the dismal outcomes of Anti-PD-1 therapy (Trujillo et al., 2019). As PTEN deletion leads to PI3K activation, treatment of mouse models of melanoma with selective PI3Kβ inhibitors improved the efficacy of Anti-PD-1 and Anti-CTLA-4 antibodies. In addition, in glioma patients treated with Anti-PD-1 antibodies (Nivolumab or Pembrolizumab), genomic and transcriptional analysis showed that PTEN loss of function mutations in tumors were significantly increased in non-responders (Zhao et al., 2019). In summary, these findings suggest that PTEN deletion enhances tumor resistance to ICB and supports the idea that ICB combined with PI3K-AKT pathway inhibitors may benefit cancer patients bearing PTEN deletion (Peng et al., 2016).

#### Activation of MAPK Signal Pathway

MAPKs are often activated in human cancer, leading to cell proliferation, and other malignant phenotypes. On the other hand, MAPKs are associated with tumor immune evasion. For example, B-Raf proto-oncogene (BRAF) gain of function mutation (BRAF V600E) contributes to immune escape of melanoma cells. MAPK signaling pathway can inhibit T cell immune response by enhancing the expression of immunomodulatory cytokines IL-6 and IL-10, which play a key role in cancer immune escape (Sumimoto et al., 2006). Thus, combining immunotherapy and BRAF targeted therapy may improve tumor killing. Indeed, a first-in-human clinical trial of Dabrafenib, Trametinib, and Pembrolizumab (NCT02130466) in 15 patients with BRAF-mutated metastatic melanoma found that the combination of BRAF and MEK inhibitors with PD-1 blocking therapy induced an objective responses in 11 patients (73%) (Ribas et al., 2019). MAPKs can regulate the activity of myosin II. High activities of ROCK-myosin II pathway are associated with melanoma mice resistance to MAPK inhibitors and immunotherapy. Therefore, combined use of ROCK inhibitors, targeted therapy and immunotherapy may overcome drug resistance of melanoma (Orgaz et al., 2020).

Inactivation of F-box and WD repeat domain containing seven (FBXW7) gene has been found to be associated with tumor resistance to immunotherapy. FBXW7 is a tumor suppressor gene and is the most frequently mutated member of the F-box protein family in human cancers (Yumimoto and Nakayama, 2020). In Anti-PD-1 sensitive melanoma mouse models, Fbxw7 deletion or mutation in tumor cells altered the immune microenvironment by decreasing immune cell infiltration and diminished the activation of viral sensing and interferon signaling pathways *in vivo*. It is interesting that restoration of dsRNA sensing in Fbxw7-deficient cells increased MHC-I expression and sensitized Fbxw7-deficient tumors to Anti-PD-1 (Gstalder et al., 2020). Besides, Serine/Threonine kinase 11 (STK11) mutation is the main driving factor of primary resistance of PD-1 inhibitors in lung adenocarcinoma (LUAC) with KRAS mutations (Skoulidis et al., 2018). In a mouse model of KRAS-driven NSCLC, STK11 gene deletion led to the accumulation of neutrophils with T cell-suppressive effects and increased the expression of T cell exhaustion markers and tumor-promoting cytokines (Koyama et al., 2016).

### IFN-γ Signal Pathway-Double-Edged Sword

Interferon usually activates the immune system to protect cells from virus infection. However, interferon also suppresses the immune system. Interferon-γ (IFN-γ), produced by tumor-specific T cells, can recognize the corresponding receptors on tumor cells or antigen-presenting cells to exert an effective anti-tumor immune response. IFN-γ can enhance the expression of MHC molecules, thus enhancing tumor antigen presentation. IFN-γ can also affect other immune cells, or directly inhibit the proliferation of tumor cells and promote their apoptosis. IFN-γ produced by tumor infiltrating T cells leads to blood flow arrest and subsequent tumor collapse by inducing tumor vascular degeneration (Kammertoens et al., 2017). In a cohort of 12 melanoma patients who were ineffective to Ipilimumab therapy, exon sequencing showed that there were copy number alteration (CNA) in IFN-g pathway genes. The most significant CNA included the genome loss of key IFN-g pathway genes, such as IFNGR1, IRF1, JAK2, and IFNGR2, as well as the amplification of important IFN-g pathway inhibitors including SOCS1 and PIAS4 (Gao et al., 2016). Therefore, mutations and deletions of IFN γ pathway-related proteins in tumor cells, such as IFNGR1 and IFNGR2, JAK1 and JAK2, STATs and IRF1, would lead to drug resistance to ICB (Shin et al., 2017). Paradoxically, persistent interferon signals can cause T cell depletion and immunosuppression. Long-term IFN-β transcription was observed in immunotherapy-resistant tumors. IFN-β accumulation could induce PD-L1 and NOS2 expression in both tumor and DCs, which is associated with intratumor accumulation of regulatory T cells (Tregs) and myeloid cells and acquired resistance to Anti-PD-1 monoclonal antibody (Jacquelot et al., 2019). Long-term interferon signals were observed to coordinate both PD-L1-dependent and PD-L1-independent resistance to ICB-involved therapy such as radiation plus anti-CTLA-4 in melanoma mouse models and patients. Persistent type II interferon signals allow tumors to obtain STAT1-related epigenetic changes and enhance the expression of interferon-stimulating genes (ISGs) and ligands of multiple T cell inhibitory receptors (Benci et al., 2016). In summary, the role of IFNs in tumor immune response is a double-edged sword. In the initial stage, IFNs can activate dendritic cells to promote the cross-activation of tumor-specific CD8^+^ T cells, but persistent IFNs can produce a negative feedback effect, resulting in T cell depletion and immunosuppression (Benci et al., 2019).

### Innate Anti-PD-1 Resistance Signature

The transcriptional characteristics of 15 cases of reactive and 13 cases of non-reactive tumors were analyzed before Anti-PD-1 treatment. Analysis of differentially expressed genes (DEGs) showed that mesenchymal transformation genes (AX1, ROR2, WNT5A, LOXL2, TWIST2, TAGLN, FAP), immunosuppressive genes (IL10, VEGFA, VEGFC) and monocyte/macrophage chemoattractant genes (CCL2, CCL7, CCL8, and CCL13) were highly expressed in non-reactive preconditioned tumors (Hugo et al., 2017). In addition to mesenchymal genes, genes related to wound healing and angiogenesis are also considered to be T cell suppressor genes (Motz and Coukos, 2011). A group of 26 transcriptomic signatures were co-enriched en bloc in 9 of 13 non-response vs. 1 of 15 response pre-Anti-PD-1 tumors. Co-enrichment of these signals are collectively referred to as the innate Anti-PD-1 resistance (IPRES) (Hugo et al., 2017). Therefore, the expression analysis based on a single gene shows that interstitial and T cell inhibitory inflammation or angiogenic tumor phenotype is associated with IPRES. MAPK targeted therapy induces similar characteristics in melanoma, suggesting that non-genomic MAPK inhibitor resistance mediates cross-resistance to Anti-PD-1 therapy. The co-enrichment of IPRES signatures also defines transcriptional subsets in common human malignant tumors, such as pancreatic cancer, whose IPRES-rich transcriptional subsets constitute the majority of tumors (No authors listed, 2016).

### Increased Expression of Constitutive PD-L1

High expression of PD-L1 in tumor or tumor microenvironment can inhibit the response of anti-tumor T cells. Tumor cells resisting to PD1/PD-L1 based immunotherapy is context dependent. PD-L1 expresses either on tumor cells or stromal cells adjacent to tumor could contribute to tumor growth and resistance, especially in less immunogenic tumor cells. PD-L1 on MC38 tumor cells can directly inhibit CD8^+^ T cell responses and reduce CD8^+^ T cell cytotoxicity and the CD8^+^ ratio relative to Treg cells (Juneja et al., 2017). The expression of PD-L1 on host cells, especially tumor-associated macrophages (TAM) is also an important factor in tumor immune escape (Noguchi et al., 2017). Metastatic melanoma can release extracellular vesicles, mainly in the form of exocrine, carrying PD-L1 on its surface. When these vesicles are stimulated by IFN-γ, the number of PD-L1 increases, which inhibits the function of CD8^+^ T cells and promotes tumor growth (Chen L. et al., 2018; Xie et al., 2019). Genetic mutations within PD-L1 locus could result in overexpression of PD-L1 or stabilizing PD-L1 mRNA. Structural variations (SVs) commonly disrupting the 3′ region of the PD-L1 gene is a unique genetic mechanism of immune escape, which always lead to a significant increase of aberrant PD-L1 transcripts (Kataoka et al., 2016). Studies have shown that transcription factor YY1 is overexpressed in most tumors and participates in regulating the resistance of tumor cells to cellular immunotherapy. There are several signal crosstalk pathways between YY1 and the regulation of PD-L1 expression, including p53, miR34a, STAT3, NF-kB, PI3K/AKT/mTOR, c-Myc, and COX-2. Therefore, the role of YY1 in tumor immune resistance may be related to the overexpression of PD-L1 on cancer cells (Hays and Bonavida, 2019).

### Epigenetic Changes

DNA methylation at the CpG sites is the most common and stable epigenetic change in cancer. Hypermethylation limits immune checkpoints and blocks immunotherapy by inhibiting the endogenous interferon response. However, global DNA hypomethylation can contribute to the constitutive upregulation of PD-L1 on melanoma cells, which might contribute to resistance to immunotherapy (Jung et al., 2019). EZH2 is a histone lysine N-methyltransferase. It can inhibit gene expression at the epigenetic level by promoting histone methylation. EZH2 can also promote DNA methylation to inhibit gene transcription. Inhibition of EZH2 activity can stimulate the expression of tumor suppressor genes, thus inhibiting tumor cell growth (Emran et al., 2019). In the Squamous cell carcinoma of head and neck (HNSCC) data set of cancer genome map, the expression of EZH2 was negatively correlated with the pathway of antigen processing. In human and mouse HNSCC, EZH2 inhibitor or CRISPR-mediated EZH2 deficiency enhanced MHC-I expression and antigen presentation on tumor cells, and increased antigen-specific CD8^+^ T cell proliferation, IFN-γ production, and tumor cell cytotoxicity. In the HNSCC model of Anti-PD-1 resistance, combination therapy with Anti-PD-1 and EZH2 inhibitor could inhibit tumor growth (Zhou et al., 2020). In melanoma, fat mass and obesity-associated protein (FTO), a N-methyladenosine (mA) demethylase, plays an important role in promoting tumor resistance to Anti-PD-1. FTO promotes the progression of melanoma. PD-1 antibody does not play a role in tumors caused by B16 cells with high expression of FTO. However, FTO knockout increases MA methylation in the critical pro-tumorigenic genes and makes melanoma cells sensitive to Anti-PD-1 treatment in mice (Yang et al., 2019).

### Lack or Exhaustion of T Cells

The success of tumor immunotherapy mainly relies on T cells. The loss of T cell function will greatly weaken the efficacy of immunotherapy, which is an important reason for tumor immune drug resistance and escape. T cells become exhausted upon binding of their surface PD-1 to PD-L1. In addition, T cell exhaustion (Tex) can be caused by other co-inhibitory receptors such as CTLA-4, TIM-3, TIGIT, and LAG-3 (Schnell et al., 2020). CD28 is a T cell co-stimulatory molecule, which plays a key role in the activation and proliferation of T cells. Blocking the CD28-B7 co-stimulatory pathway suppresses the proliferation and activation of tumor-specific CD8^+^ T cells, and reduces the response to Anti-PD-1/PD-L1 therapy (Hui et al., 2017). It has been found that in the Anti-PD-1 resistance model, simultaneous Anti-PD-1 and vaccine therapy can reverse the resistance, while blocking PD-1 before antigen activation can eliminate the therapeutic effect. PD-1 blockade in unprimed or suboptimally primed CD8 cells induces resistance through the induction of PD-1^+^ CD38^+^ CD8^+^ cells that lead to incorrect T cell receptor signal transduction and non-response to antigen restimulation. On the other hand, the PD-1 blocking of the best activated CD8 cells prevents the production of dysfunctional CD8 cells, thus reversing the resistance (Verma et al., 2019). In human ovarian cancer (OvCa), PD-L1 is not highly expressed in the tumor microenvironment (TME). On the contrary, another checkpoint molecule, B7-H3, is highly expressed in both tumor cells and tumor infiltrating antigen presenting cells (APC), which is associated with T cell depletion in patients. Using the OvCa mouse model, it was found that B7-H3 expressed on tumor cells, but not on host cells, played a leading role in inhibiting anti-tumor immunity. Blocking of B7-H3, rather than blocking of PD-1, prolonged the survival time of tumor-bearing mice (Cai et al., 2020). In preclinical models and patients with melanoma, the combination of Anti-CTLA-4 and Anti-PD-1 therapy can impair the anti-tumor immunity under the condition of low tumor load. The combination therapy induces the loss of tumor-specific T cells and changes the landscape of T cell pedigree, tilting the distribution of T cells to lower-frequency clonotypes (Pai et al., 2019). Unlike Tex, memory T cell would make the landscape of TME more perfect, which is under closely observation and exploration (Mok et al., 2019).

## External Causes of Tumor Resistance to Immunotherapy

### Immunosuppressive Cells and Cytokines in Tumor Microenvironment

Tumor associated macrophages (TAMs) and macrophage colony stimulating factor (CSF-1). TAMs are a special kind of macrophages found in TME. TAMs are a large cell population in TME. Studies have shown that the infiltration of macrophages into tumors is related to the poor prognosis and drug resistance in many types of cancers. The data from a variety of mouse cancer models show that TAMs can promote tumor generation and development by promoting angiogenesis, enhancing the metastatic and invasive ability of tumor cells and inhibiting anti-tumor immunity (Cassetta and Pollard, 2018). TAMs include M1 and M2 macrophages. In most cases, M2 macrophages account for the majority of TAMs. M1 macrophages can highly express IL-12, IL-23, MHC, and B7 family molecules to promote antigen presentation and activation of Th1 cells, thus promoting anti-tumor immune responses. M2 macrophages can secrete inhibitory cytokines IL-10 and TGF-β, which can inhibit the activation and proliferation of CD4^+^/CD8^+^ T cells and promote proliferation of Tregs. At the same time, M2 macrophages further secrete a variety of chemokines such as CCL2, CCL3 to enhance the recruitment of Tregs (Ruffell and Coussens, 2015; Vitale et al., 2019). Excess osteopontin (OPN) from hepatoma cells stimulates macrophages to secrete CSF1, through PI3K-AKT-NF-κB pathway, to bind to colony-stimulating factor-1 receptor (CSF1-R) on macrophages. Furthermore, the PI3K-AKT signal pathway was activated to polarize TAMs into immunosuppressive M2 phenotype, which finally causes immune escape in liver cancer. The application of CSF1-R inhibitor can significantly inhibit the infiltration of TAMs in tumor and increase the infiltration and killing activity of CD8^+^ T lymphocytes in tumor, which reshapes TME of hepatocellular carcinoma and enhances the efficacy of PD-L1 antibody (Ramesh et al., 2019; Zhu et al., 2019).

#### Tregs and Neuropilin-1 (Nrp1)

Tregs are a T cell subset that are constituted of natural Tregs (nTregs) and induced Tregs (iTregs). Tregs are well known to maintain self-tolerance. In the process of tumorigenesis, Tregs inhibit TCR-mediated activation and proliferation of CD4^+^/CD8^+^ T cells to promote tumor immune evasion (Li and Rudensky, 2016). The transcription factor forkhead box P3 (FOXP3) is specifically expressed by Tregs, which is involved in the differentiation and development of Tregs and tumor immune escape (Sakaguchi et al., 2020; Savage et al., 2020). It has been found that a surface protein called Nrp1 is expressed in almost all Tregs infiltrating into mouse tumors. In TME, Nrp1 is very important for maintaining the function, integrity and survival of Tregs and thus functions to suppress anti-tumor immune response (Mariathasan et al., 2018). Treg-specific Nrp1 deletion has been shown to significantly dampen tumor growth. High Nrp1^+^ Tregs have been found to correlate with poor prognosis in cancer patients. On the other hand, IFN-γ functions to weaken Tregs function, by making Tregs “fragile,” and is thus critical to the success of PD-1 blockade (Overacre-Delgoffe et al., 2017).

#### Myeloid-Derived Suppressor Cells (MDSCs)

MDSCs, a group of heterogeneous cells derived from bone marrow, are the precursors of DCs, macrophages and/or granulocytes and have the ability to significantly inhibit immune responses (Talmadge and Gabrilovich, 2013). The inhibitory activity of MDSCs is regulated by the cellular stress sensor C/EBP homologous protein (CHOP). CHOP deletion has been shown to decrease the immunomodulatory function of MDSCs and hence activate T cell function and induce anti-tumor responses (Thevenot et al., 2014). In a mouse model of prostate cancer, MDSCs (CD11b^+^ Gr1^+^) have been found to promote tumor development. In a chimera mouse model of metastatic castration-resistant prostate cancer (mCRPC), primary and metastatic CRPC show strong synergistic response when ICB is combined with MDSC targeted therapy (Lu et al., 2017). Characterization of several preclinical tumor models and clinical specimens found that while CD8^+^ T cells were activated by PD-1 blocking, these CD8^+^ T cells induced PD-L1-NLRP3 inflammatory signal cascades in tumor cells, resulting in recruitment of granulocytic MDSCs (PMN-MDSCs) into tumor tissue, thus inhibiting anti-tumor immune response. The genetic and pharmacological inhibition of NLRP3 inhibited the tumor infiltration of PMN-MDSCs and significantly enhanced the efficacy of Anti-PD-1 immunotherapy (Theivanthiran et al., 2020). A recent study found that mouse and human PMN-MDSCs express high fatty acid transporter 2 (FATP2). FATP2 deletion resulted in a loss of inhibitory activity of PMN-MDSCs. Selective pharmacological inhibition of FATP2 could inhibit the activity of PMN-MDSCs and significantly delayed tumor progression. Combined with ICB, inhibition of FATP2 could prevent tumor development in mice (Veglia et al., 2019).

#### Transforming Growth Factor-β (TGF-β)

TGF-β has a variety of biological functions and plays an important role in regulating cell growth, proliferation, differentiation, apoptosis, migration, and immunity. Most advanced tumor cells can secrete TGF-β. Once the level of TGF-β increases, it can block the differentiation of immature T cells into Th1 cells and promote the transformation of immature T cells into Tregs. In addition, it inhibits antigen presentation of DCs, resulting in immune escape of tumor cells (Batlle and Massagué, 2019). A TGF-β inhibitor could promote immune cell infiltration into tumor, effectively preventing tumor metastasis. Combination of TGF-β inhibitors and immunotherapy can enhance the anti-tumor effect of TGF-β inhibition and significantly increase the survival rate of tumor-bearing mice (Tauriello et al., 2018). The lack of response to Anti-PD-L1 antibody (Atezolizumab) in a patient with metastatic urothelial cancer was found to be related to TGF-β signaling in fibroblasts that might be linked to CD8^+^ T cell exclusion from the tumor parenchyma. Using a mouse model of immune rejection, it was found that combined use of TGF-β blockers and Anti-PD-L1 antibodies promoted the infiltration of T cells into the tumor center, which stimulates strong anti-tumor immunity and tumor regression (Mariathasan et al., 2018).

#### Angiogenic Factor

Most solid tumors exhibit vascular abnormalities. Impaired vascular perfusion and increased vascular permeability promote tissue hypoxia, acidosis and necrosis, inhibiting the function of effector T cells, which contributes to immune escape. Vascular abnormalities result from elevated levels of pro-angiogenic factors, such as vascular endothelial growth factor (VEGF) and Angiopoietin-2 (ANG2). Treatment with anti-vascular drugs targeting these pro-angiogenic factors can normalize abnormal tumor vessels, which in turn increases the infiltration of immune effector cells and transforms the inherent immunosuppressive TME into immune-supported microenvironment (Fukumura et al., 2018). ICB can also promote the normalization of tumor vessels. Initial vascular normalization reduces the immunosuppressive process in the tumor microring and promotes the infiltration of effector T cells as well as improves their function, leading to a further normalization of tumor blood vessels. This feedback loop between immune reprogramming and tumor vascular normalization has a self-reinforcing effect and ultimately promotes immune-mediated tumor elimination (Huang et al., 2018; Liu et al., 2019).

#### Tumor Necrosis Factor (TNF)

Like interferon-γ, TNF is secreted by activated T cells. TNF-mediated bystander killing maybe underlie the effects of T cells on antigen-negative tumor cells. In this context, tumor immune escape can be caused by a loss of sensitivity to TNF (Kearney et al., 2018). The loss of sensitivity of tumor cells to TNF could be mediated by TRAF2, given that inhibition of TRAF2 in tumor cells re-sensitizes them to TNF *in vitro* and improves the efficacy of ICB in mice bearing ICB-resistant tumors (Vredevoogd et al., 2019).

#### Proinflammatory Cytokines Interleukin

Targeting a proinflammatory cytokine interleukin 6 (IL6) increases responses of tumor-specific Th1 and subsequent anti-tumor effects in tumor-bearing mice. Blockade of IL-6 can upregulate the expression of PD-L1 in melanoma cells. Combined blockade of IL6 and PD-1/PD-L1 signals increases the expression of T cell-attracting chemokines and facilitates IFN-γ-producing CD4 T cell infiltration into tumor tissues, producing a synergistic anti-tumor effect (Tsukamoto et al., 2018). Mechanistically, IL-6/JAK1 pathway promotes PD-L1 phosphorylation, which seems to be the main driving factor of cancer immune escape in mouse model of liver cancer. As a result, blocking IL-6 pathway can prolong the effects of immunotherapy for liver cancer (Chan et al., 2019).

### Tumor Metabolites in Immune Microenvironment

Immunosuppressive metabolites in TME can inhibit anti-tumor immunity, which is attributable to inhibition of immune cell infiltration. Metabolic disorders of cancer cells further affect the expression of cell surface markers, thus interfering immune monitoring.

#### Aerobic Glycolysis

Unlike normally differentiated cells which mainly rely on mitochondrial oxidative phosphorylation to produce energy, even under aerobic conditions, cancer cells are prone to use glycolysis to provide energy, the phenomenon known as the Wahlberg effect. Transcription factor SIX1 directly increases the expression of many glycolysis genes, promoting Warburg effect and tumor growth (Li L. et al., 2018). In mouse models of triple negative breast cancer (TNBC), restricted glycolysis inhibited the expression of tumor granulocyte colony stimulating factor (G-CSF) and granulocyte macrophage colony stimulating factor (GM-CSF), reducing MDSCs and enhancing anti-tumor immunotherapy. Tumor glycolysis coordinates the molecular network of AMPK-ULK1, autophagy and CEBPB pathways to affect MDSCs and maintain tumor immunosuppression (Li W. et al., 2018). Histone lysine (K)-specific methyltransferase 2D (KMT2D) inhibitor increased aerobic glycolysis and changed the liposome characteristics of pancreatic cancer cells. Therefore, KMT2D may play a new previously unknown anti-tumor effect by regulating glucose/fatty acid metabolism in pancreatic cancer (Koutsioumpa et al., 2019).

#### Adenosine

Adenosine, a ligand for adenosine 2A receptor (A2AR) on immune cells, acts to suppress anti-tumor immunity in TME. Adenosine is produced by CD39 and CD73 expressed on vascular endothelial cells and Tregs. The expression of CD39 on the surface of Tregs could inhibit the anti-tumor immunity mediated by natural killer (NK) cells *in vitro* and *in vivo* (Sun et al., 2010). CD39 deletion strongly inhibited tumor growth in the liver in a mouse model of metastatic melanoma. Tumor initiation, growth and metastasis can be limited by co-blocking CD73 and A2AR, using compound gene-targeted mice or therapeutic drugs (Young et al., 2016). A first human study of A2AR antagonists for cancer treatment has confirmed that combined A2AR antagonists and Anti-PD-L1 has anti-tumor activity in patients with refractory renal cell carcinoma, correlating with increased tumor infiltration of CD8^+^ T cells (Fong et al., 2020). It has been shown that tumor cells treated with PD-L1 blocking antibody produce adenosine by up-regulating CD38 and thus inhibit the function of CD8^+^ T cells, leading to acquired drug resistance (Chen L. et al., 2018). It was found that inhibition of adenosine A1 receptor, ADORA1, can upregulate the expression of PD-L1 in melanoma cells, leading to CD8^+^ T cell depletion and tumor immune evasion. Retrospective analysis showed that melanoma patients bearing low ADORA1, high ATF3 or high PD-L1 in their tumor tissues had higher response rates to Anti-PD-1 monoclonal antibody and better prognosis (Liu et al., 2020).

#### Arginine/Tryptophan

Arginine and tryptophan are essential amino acids for the proliferation and differentiation of T cells. Arginase 1 (ARG1) is an enzyme that catalyzes the degradation of arginine and indoleamine 2-dioxygenase 1 (IDO) and tryptophan 2-dioxygenase (TDO) catalyzes the degradation of tryptophan. The increase of immunomodulatory cells expressing ARG1 including M2 TAMs, immune tolerant DCs and Tregs in TME can inhibit anti-tumor immunity by degrading arginine and limiting the utilization of this amino acid by T cells (Lemos et al., 2019). In addition, IDO and TDO decompose tryptophan (Trp) into kynurenine (Kyn). The accumulation of Kyn can increase the number of peripheral Treg cells and decrease the proliferation of effector T cells (Cervenka et al., 2017). After a comprehensive analysis of the serum metabolites of patients with advanced melanoma and renal cell carcinoma treated with Anti-PD-1 antibody (nivolumab), it was found that the increase of serum Kyn/Trp ratio was an adaptive mechanism of drug resistance, which was correlated with the poor overall survival rate (Li H. et al., 2019). Kyn can be degraded into immune inert, non-toxic and easy-to-remove metabolites by a drug-optimized PEGylated kynureninase (PEG-KYNase). PEG-KYNase treatment increases CD8^+^ T cell proliferation and infiltration in tumor. PEG-KYNase combined with ICB is effective in the treatment of melanoma, breast cancer or colon cancer (Triplett et al., 2018).

#### Fatty Acids and Cholesterol

A number of studies have shown that lipids accumulate in macrophages, DCs and MDSCs in tumors. In PMN-MDSCs, fatty acid transport protein 2 (FATP2) can promote the utilization of arachidonic acid and the synthesis of prostaglandin E2, mediating immunosuppressive function. FATP2-deficient mice displayed smaller tumors than FATP2 -proficient mice. Lipofermata, an inhibitor of FATP2, has a significant inhibitory effect on subcutaneous tumors in immunocompetent mice (Wellenstein and de Visser, 2019). The concentration of lipids and the expression of prostaglandin E2 (PGE2) and FATP2 in peripheral blood of patients with head and neck cancer, breast cancer and lung cancer were higher than those in healthy subjects. Inhibition of cholesterol esterification in T cells by gene ablation or drug inhibition of cholesterol esterification key enzyme ACAT1 can enhance the proliferation and effector function of CD8^+^ T cells. ACAT1-deficient CD8^+^ T cells are superior to wild type CD8^+^ T cells in controlling the growth and metastasis of mouse with melanoma. ACAT1 inhibitor (Avasimibe) showed a good anti-tumor effect in melanoma bearing mice (Yang et al., 2016). Furthermore, HCD or ApoE gene deletion elevated serum levels of cholesterol and inhibited tumor growth in mice injected with hepatoma cells or chemical carcinogens. Cholesterol has been found to accumulate in NK cells to promote NK cell-mediated killing of hepatoma cells (Qin et al., 2020).

### New Immune Checkpoints

In addition to the well-recognized immune checkpoints PD-1/PD-L1 and CTLA-4, more and more studies have found that tumor immune escape and drug resistance may be related to other less-appreciated immune checkpoints (Andrews et al., 2019).

#### TIM-3

T cell immunoglobulin and mucin domain-containing protein 3 (TIM-3) is a member of the TIM family of immunomodulatory proteins. In humans, members of the TIM family are encoded by three genes, of which HAVCR1 encodes TIM-1, HAVCR2 encodes TIM-3, and TIMD4 encodes TIM-4. TIM-3 plays a role in transplant tolerance and autoimmunity, mycobacterium tuberculosis infection, chronic viral infection and cancer. In cancer, TIM-3 marks the most dysfunctional subgroup of tumor infiltrating CD8^+^ PD-1^+^ T cells. Blocking TIM-3 and PD-1 antibodies have a synergistic effect on improving tumor antigen-specific CD8^+^ T cell responses and inhibiting tumor growth. TIM-3^+^ Treg cells are the main Treg cells group in tumors, so TIM-3^+^ Treg cells may also be targeted by TIM-3 antibodies (Starling, 2017). Galectin-9 is the main inhibitory ligand of TIM-3, and TIM-3-galectin-9 interaction plays an important role in suppressing immune response. In patients with metastatic NSCLC, it was found that the accumulation of bone marrow-derived suppressor cells expressing TIM-3 or galectin-9 was related to tumor resistance to Anti-PD-1 antibody. Moreover, Anti-TIM-3 blocking antibody reverses tumor resistance to PD-1 blockade in patients with lung cancer (Limagne et al., 2019).

#### LAG-3

Lymphocyte activation gene-3 (LAG-3) is a type I transmembrane protein composed of 498 amino acids, which is selectively expressed on activated T cells, NK cells, and DCs. LAG-3 protein is composed of four extracellular immunoglobulin superfamily (IgSF)-like domains (D1-D4). Its regulatory function on T cells is similar to that of PD-1, mainly as a receptor for the delivery of inhibitory signals (Lui and Davis, 2018). Fibrinogen-like protein 1 (FGL1) is the main inhibitory ligand of LAG-3. Both anti-FGL1 and anti-LAG3 could promote anti-tumor T cell immunity. It was found that the anti-tumor effect of anti-FGL1 monoclonal antibody depended on LAG-3 and anti-LAG-3 depended on FGL1 rather than MHC-II or other ligands. Compared with normal tissue, human solid tumors including lung cancer, prostate cancer, melanoma and colorectal cancer showed higher FGL1 expression. FGL1-LAG-3 interaction is another tumor immune escape pathway independent of B7-H1-PD-1 pathway. Blocking this pathway has a synergistic effect with Anti-PD-1 therapy (Wang et al., 2019).

#### NKG2A

CD94/NKG2A, A Natural Killer cell inhibitory receptor heterodimer molecule, can inhibit CD8^+^ T cell function, and blocking NKG2A enhances the immune activity of NK cells and T cells in mice, thus improving the anti-tumor immune responses. The ligand of NKG2A is a HLA-E protein expressed on the surface of tumor cells. Monalizumab, a humanized monoclonal antibody against NKG2A, only marginally stimulates anti-tumor immune responses. However, combined Monalizumab and ICB treatment achieves optimal therapeutic index. In a phase II clinical trial conducted in 31 patients with squamous cell head and neck cancer, the combination of Monalizumab and Cetuximab reached an objective remission rate (ORR) of 31%. Thus, NKG2A seems to be a potential new immunotherapy checkpoint (André et al., 2018; van Montfoort et al., 2018).

#### TIGIT

T-cell immunoglobulin and ITIM domain (TIGIT), a member of the PVR/nectin family, is an immune checkpoint that negatively regulates T cell function. TIGIT is highly expressed on effector T cells and Treg cells. It has been found that the progression of multiple myeloma (MM) in mice and humans is related to high expression of TIGIT on CD8^+^ T cells. Blocking TIGIT with monoclonal antibody enhanced the effector function of CD8^+^ T cells in MM patients and inhibited the development of MM (Guillerey et al., 2018). TIGIT also expresses on NK cells and leads to NK cell depletion. In a variety of tumor models, it was found that compared with NK cells around tumor, NK cells in tumor core express higher TIGIT, resulting in a significant decrease in anti-cancer factors such as IFN-γ and TNF produced by NK. Blocking TIGIT by genetic targeting or blocking antibodies could effectively inhibit tumor growth in mice and significantly prolong the survival of tumor-bearing mice (Zhang et al., 2018). Other newly discovered immune checkpoints include ADAR1 (Ishizuka et al., 2019), PBRM1 (Braun et al., 2019), ATM (Zhang et al., 2019), PTPN2 (Manguso et al., 2017), CD47 (Li et al., 2020), and CD24 (Bradley, 2019). Along with TIGIT, these immune checkpoints may cause tumors to resist immunotherapy by inhibiting the activity of T cells and/or NK cells.

### Intestinal Microflora

In the human intestinal tract, there is a group of active microorganisms that live in symbiosis with the host, which is what we call the intestinal microbiota. As a complex microecosystem in the human body, intestinal microbiota plays an extremely important role in maintaining the normal immune defense function of the body. At the same time, it is closely related to many metabolic diseases, immune diseases, and tumors. It has been found that intestinal flora has a great influence on the effect of tumor immunotherapy. Intestinal microflora disorders weaken the response of tumors to ICB, causing primary tumor resistance to immunotherapy. Intestinal microflora disorders include significant inhibition of Lactobacillus, Bifidobacterium, and Enterococcus, and significant growth of Escherichia coli and strange bacteria. In 283 patients with NSCLC, melanoma and other tumors, the use of antibiotics before immunotherapy was associated with poor overall survival (Pinato et al., 2019). In a follow-up cohort of 249 patients with lung, kidney and other cancers, 60 patients treated with antibiotics were more likely to relapse and had a shorter survival time after immunotherapy. When the feces of cancer patients showing good immune response were transplanted into aseptic mice, immune response in the recipient mice was significantly better than that in non-transplanted mice. Optimal immune response was observed in non-transplanted mice after immunization with Akkermansia muciniphila probiotics (Routy et al., 2018). A mixture of 11 bacterial strains was found to activate the immune system and slowed the growth of melanoma in mice (Li Y. et al., 2019). An immunotherapy based on flora is designed by computer modeling and engineering bacteria modification, which opens a door for immunotherapeutic drug design based on microorganisms and metabolites (Skelly et al., 2019).

## Conclusion

Primary and acquired resistance become a major challenge in the field of tumor immunotherapy. Satisfactory efficacy of immunotherapy cannot be achieved without clarifying the mechanisms of tumor immune evasion and immune drug resistance. In this review, we cataloged a variety of factors that may affect the efficacy of anti-tumor immunotherapy. We discussed a number of internal and external causes of tumor resistance to ICB, and summarized the research progress in exploiting these mechanistic factors to re-sensitize tumors for ICB.

As immunotherapy enters a new era, it is crucial to find effective biomarkers to predict the response rate of cancer patients to immunotherapy. Through continuous screening and development of biomarkers related to immunotherapy, including markers for responding groups and markers for poorly responding groups, the incidence of drug resistance in immunotherapy can be reduced and its efficiency can be improved. In addition, it appears that combination therapy would be an effective way to overcome tumor immune drug resistance. Currently, there are many combination strategies are under development, such as the combination of different immunosuppressants, costimulatory factor agonists, molecular targeting drugs, anti-vascular therapy, radiotherapy, and chemotherapy, intestinal probiotics, and the combination of epigenetic modification factors, which have achieved some good results in clinical research. Moreover, personalized immunotherapy stands for an idea approach to conquer immunotherapeutic drug resistance. Developing immunotherapeutic drugs (e.g., oncolytic virus, tumor vaccine, adoptive immune cell therapy) based on individual TME hinges on the identification of patient-specific tumor antigens (Liu and Guo, 2018).

## Author Contributions

ML put forwarded the conception and design. QH drafted the manuscript. QH, YL, and XL collected, analyzed the data, and prepared the figures. FG and ML completed critical comments and revision. All authors contributed to the article and approved the submitted version.

## Conflict of Interest

The authors declare that the research was conducted in the absence of any commercial or financial relationships that could be construed as a potential conflict of interest.
